# Effects of 2′‐fucosyllactose on the viability of starter cultures and *Bifidobacterium* strains of human origin in yogurt during refrigerated storage

**DOI:** 10.1111/1750-3841.16996

**Published:** 2024-04-05

**Authors:** Zifan Xie, Chanwoo Kim, Michael J. Miller, Yong‐Su Jin

**Affiliations:** ^1^ Food Science and Human Nutrition University of Illinois Urbana‐Champaign Urbana Illinois USA; ^2^ Fermented and Processed Food Science Division National Institute of Agricultural Sciences Wanju South Korea; ^3^ Carl R. Woese Institute for Genomic Biology, University of Illinois Urbana‐Champaign Urbana Illinois USA

**Keywords:** 2′‐fucosyllactose, bifidobacteria, fermentation, viability, yogurt

## Abstract

**Abstract:**

2′‐Fucosyllactose (2′‐FL) is postulated to provide health benefits and promote the growth of probiotics. This work was undertaken to study the effects of 2′‐FL on the viability of starter cultures and *Bifidobacterium* strains of human origin in yogurt during refrigerated storage. Yogurts were produced containing 2′‐FL (0 or 2 g/L) and *Bifidobacterium* strains of human origin (*Bifidobacterium longum* subsp. *longum* BB536 or *Bifidobacterium longum* subsp. *infantis* ATCC 15697) at a concentration of at least 10^9^ CFU/mL. All yogurts were stored at 4°C for 5 weeks. Results showed that 2′‐FL was stable in yogurts for at least 5 weeks of cold storage, and the addition of 2′‐FL did not significantly alter yogurt fermentation parameters, associated metabolites, and the viability of mixed yogurt starter cultures and *Bifidobacterium* strains (*p *> 0.05). The addition of bifidobacteria had a negative impact (*p* < 0.05) on the survival rate of starter cultures, *Streptococcus thermophilus* and *Lactobacillus delbureckii* subsp*. bulgaricus*. Meanwhile, it is difficult to maintain a high survival rate of bifidobacteria in final yogurt products, and the addition of 2′‐FL could not enhance the viability of bifidobacteria. *B. longum* BB536 survived at a level higher than 10^6^ CFU/g for 28 days, while *B. infantis* ATCC15697 maintained this level for only 7 days. In summary, this study has shown the impact of 2′‐FL and bifidobacterial species on yogurt properties, and results suggest that it is promising to use 2′‐FL in yogurt products as a prebiotic.

**Practical Application:**

Yogurt is known for its beneficial effects on human health and nutrition. This study reported the production of symbiotic yogurt containing bifidobacteria and 2′‐fucosyllactose (2′‐FL) as a functional food for specified health uses. The viability of yogurt starter cultures and probiotic bifidobacterial strains was analyzed in this study. Moreover, this research demonstrated that 2′‐FL could be added to yogurt without affecting the characteristics of yogurt significantly.

## 1 INTRODUCTION

2′‐Fucosyllactose (2′‐FL) is the most abundant human milk oligosaccharide (HMO) in both colostrum and mature milk, with concentrations of 0.76 to 4.78 g/L (Thurl et al., 2017). It is one of the most promising prebiotic oligosaccharides in HMO and has gathered great interest not only for health benefits but also for commercial applications (Zhu et al., 2022). Prebiotics are described by ISAPP (International Scientific Association for Probiotics and Prebiotics) as “a substrate that is selectively utilized by host microorganisms conferring a health benefit” (Gibson et al., 2017). As a prebiotic, 2′‐FL is postulated to provide health benefits and promote the growth of beneficial bacteria. In recent years, 2′‐FL has been produced industrially and successfully introduced into different infant formula products by various producers worldwide. However, due to the difficulty of large‐scale production and its current high cost, 2′‐FL is still rarely used in other food products such as yogurt, and its impact on food systems has not been evaluated.

Probiotics are “live microorganisms that, when administered in adequate amounts, confer a health benefit on the host”(Hill et al., 2014). Bifidobacteria are well‐known probiotic microorganisms, and their beneficial effects on human health have been the subject of many investigations (Hidalgo‐Cantabrana et al., 2017; Russell et al., 2011). Now, bifidobacteria are used globally as probiotics in various food products including yogurt, milk, infant formula, and dietary supplements (Linares et al., 2017; Prasanna et al., 2014). However, in yogurt products, the survival of *Bifidobacterium* strains is quite low, and reduced viability of probiotic bacteria in yogurt has been extensively reported (Amakiri & Thantsha, 2016; Kamel et al., 2021; Lucas et al., 2004). *Bifidobacterium animalis* subsp. *lactis*, which is animal origin, is the most commonly used *Bifidobacterium* strain in yogurt products as it has the greatest resistance to environmental stress (e.g., acidity and oxidative stress), and it is more stable in yogurt than *Bifidobacterium longum*, *Bifodobacterium breve*, and *Bifidobacterium bifidum*, which are bifidobacterial strains isolated from humans (Jayamanne & Adams, 2006). Although *Bifidobacterium* strains of human origin tend to be more sensitive to conditions related to food applications, the health benefits of human bifidobacterial species have been studied more thoroughly than those of animal bifidobacterial species (Hidalgo‐Cantabrana et al., 2017; Wong et al., 2019).

Maintaining high viable probiotic counts in food products during the entire product shelf life is important. It is recognized that the viable probiotic microorganisms should be at least 10^6^ colony‐forming units (CFU) per gram of yogurt (CFU/g) throughout the product shelf‐life to provide health benefits (Terpou et al., 2019). Various studies have been taken to enhance the viability of bifidobacteria in yogurt. Some studies have reported that reducing post‐acidification could prolong the viability of *B. breve* in yogurt during refrigerated storage (Kailasapathy et al., 2008; Ongol et al., 2007). Lower fermentation temperature can also improve the survival of bifidobacteria in the yogurt (Abe et al., 2009). Meanwhile, various ingredients such as whey protein, fructooligosaccharide, starch, and inulin are effective in maintaining the viability of bifidobacteria in yogurt (Akalin et al., 2007; Kamel et al., 2021; Rosburg et al., 2010).

The effect of prebiotic 2′‐FL on the viability of both traditional yogurt starter cultures and probiotic *Bifidobacterium* strains in yogurt has not been studied. Therefore, in this study, we evaluated yogurt fermentation parameters, changes in pH, viability of mixed yogurt starter cultures and *Bifidobacterium* strains, and changes in substrates and metabolites in yogurt products during refrigerated storage.

## 2 MATERIALS AND METHODS

### 2.1 Yogurt starters and probiotic strains

Freeze‐dried cultures of *Streptococcus thermophilus* and *Lactobacillus delbureckii* subsp*. bulgaricus* (YF‐L706, Chr. Hansen, Milwaukee, WI, USA) were stored at −80°C and defrosted at room temperature for 10 min before use. Prior to yogurt production, both *Bifidobacterium longum* subsp. *longum* BB536 and *Bifidobacterium longum* subsp. *infantis* ATCC 15697 were inoculated and cultured anaerobically (90% N_2_, 5% CO_2_, and 5% H_2_) in de Man Rogosa and Sharpe (MRS) broth (Hardy Diagnostics, Santa Maria, CA, USA) supplemented with 0.22 µm‐filter sterilized 0.05% w/v L‐cysteine (Sigma‐Al, St. Louis, MO, USA) for 24 h at 37°C for two consecutive passes.

### 2.2 Carbohydrate utilization

After two consecutive passes, stationary phase cultures of *B. longum* BB536 and *B*. *infantis* ATCC 15697 were harvested by centrifugation (3900 rpm, 10 min) (Centrifuge 5801 R, Eppendorf, Hamburg, Germany), washed twice with phosphate buffer solution (PBS) (pH 7.4), and resuspended in sugar‐free MRS (US Biological, Salem, MA, USA). Cells were inoculated 1% into sugar‐free MRS containing water, 10 g/L glucose, 10 g/L lactose, 10 g/L galactose, 10 g/L fucose (Sigma‐Aldrich), or 10 g/L 2′‐FL (AP Technology, Suwon, Korea) for 24 h at 37°C anaerobic chamber. The concentrations of substrates and metabolites at 24 h were measured using high‐performance liquid chromatography (HPLC). All media broth was supplemented with 0.05% L‐cysteine. OD_600_ was measured by a photometer (YSI 9500 photometer, YSI Inc., Yellow Springs, OH, USA). There are three independent replications for each carbohydrate.

### 2.3 Yogurt manufacturing

Milk was reconstituted (15% w/v) from skimmed milk powder (Sigma‐Aldrich). Yogurt treatments containing 2′‐FL received a final concentration of 2 g/L 2′‐FL. The ingredients were combined, homogenized, and heated at 85°C for 30 min. The yogurt milk was then cooled to 43°C and inoculated with yogurt starter culture (0.02%). Overnight cultures of the bifidobacteria strains, *B. longum* BB536 and *B. infantis* ATCC 15697, were centrifuged (3900 rpm, 5 min). The media was decanted, and the pellets were washed twice with PBS buffer. The pellets were added, individually, to each yogurt treatment to obtain a concentration of at least 10^9^ CFU/mL. Each treatment was divided into 1‐mL aliquots and fermented at 42°C until pH 4.4 was reached. There were three replications for each treatment.

When yogurt fermentation was completed, the samples were cooled and then stored in a refrigerator (4°C) for 5 weeks. Samples were collected prior to fermentation (day 0), the day following fermentation (day 1), and once every week for 5 weeks (days 7, 14, 21, 28, and 35), for microbiological, acidification, and HPLC analyses. Each sample was from a previously undisturbed yogurt.

### 2.4 pH‐value

The pH values of the yogurt samples were measured at room temperature using a digital pH meter (Accumet AR15, Fisher Scientific, Pittsburgh, PA, USA). During yogurt fermentation, the pH values were measured every hour until pH 4.4 was reached. Then, the pH values were measured at each time point (day 1, 7, 14, 21, 28, and 35) during cold storage, and each sample was from a previously undisturbed yogurt.

### 2.5 Culture viability determination

To determine cell viability, each sample was serially diluted in PBS buffer, plated in duplicate on modified Reinforced Clostridial Agar (RCA) plates (Sigma‐Aldrich) containing X‐α‐Gal (5‐bromo‐4‐chloro‐3‐indolyl‐ α‐galactopyranoside) (Gold Biotechnology, Inc., St. Louis, MO, USA), and incubated anaerobically at 37°C for 48 h. *Bifidobacterial* (blue) and yogurt culture (white) colonies were counted (Rosburg et al., 2010). Because of the presence of yogurt starter cultures, it was hard to monitor bifidobacteria culture survival below 10^6^ CFU/g.

### 2.6 Quantification of metabolites using HPLC

Aliquots (1 g) of each yogurt sample were individually homogenized with 1 mL of mobile phase for 5 min in a vortex. The homogenates were centrifuged at 14,000 x *g* for 15 min (Centrifuge 5424 R, Eppendorf, Hamburg, Germany) at 4°C to remove the solid mass. After centrifugation, the supernatants were filtered through 0.45‐µm membranes. Samples were stored at −20°C before analysis. Analytical standards of 2′‐FL (AP Technology), lactose, galactose, glucose, fucose, lactate, acetate, and ethanol (Sigma‐Aldrich) were used to generate standard curves. HPLC analytical method was modified from the previous method (Shin et al., 2020). All experiments were performed in triplicate. The retention time for 2′‐FL was 6.70 min, lactose was 7.31 min, glucose was 8.56 min, galactose was 9.18 min, fucose was 10.75 min, lactate was 11.97 min, acetate was 14.69 min, and ethanol was 21.14 min. The concentrations of substrates and metabolites in culturing media or yogurt samples were quantified using HPLC (Agilent 1200 Series, Agilent Technologies, Santa Clara, CA, USA) equipped with a Rezex ROA‐Organic Acid H+ (8%) column (Phenomenex Inc., Torrance, CA, USA) and a refractive index detector. The column was eluted with 0.005 N of H_2_SO_4_ at a flow rate of 0.6 mL/min at 50°C. The results were used to calculate the concentrations of metabolisms in the yogurt samples (mg/g, mg per gram of yogurt).

### 2.7 Statistical analysis

The results were reported as the means of independent experiments ± standard deviation. Statistical significance was determined by analysis of variance (ANOVA). Distinct superscript letters were employed to denote significant differences in tables. *p* values less than 0.05 were considered to indicate statistically significant differences. Graphs were plotted using the GraphPad prism.

## 3 RESULTS AND DISCUSSION

### 3.1 Carbohydrate utilization of *B. longum* BB536 and *B. infantis* ATCC 15697

Two *Bifidobacterium* strains of human origin were selected for this study, *B. longum* BB536 and *B. infantis* ATCC 15697, both of which are well‐studied for their health benefits (Underwood et al., 2015; Wong et al., 2019). To better understand the sugar utilization and product formation profiles of *B. longum* BB536 and *B. infantis* ATCC 15697 in yogurt samples, we first studied their growth patterns in pure culture media. We cultured these two strains in sugar‐free MRS broth supplemented with 10 g/L lactose, glucose, galactose, fucose, or 2′‐FL. Growth curves and substrates and metabolites concentration after 24 h of incubation were measured.

**FIGURE 1:**
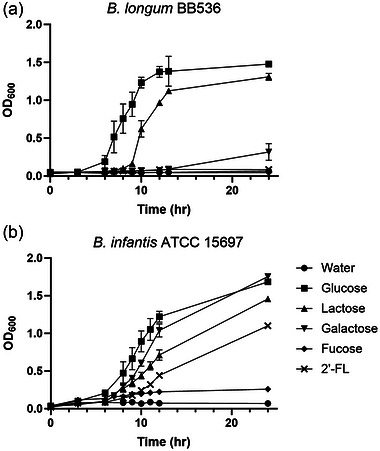
The growth curves of *Bifidobacterium longum* subsp*. longum* BB536 (A) and *Bifidobacterium longum* subsp*. infantis* ATCC 15697 (B) in sugar‐free de Man, Rogosa and Sharpe (MRS) media with different carbohydrate sources (10 g/L glucose, 10 g/L lactose, 10 g/L galactose, 10 g/L fucose, and 10 g/L 2′‐fucosyllactose [2′‐FL]). Results are the average of biological replicates (*n* = 3). Error bars represent standard deviations (SD) and are not displayed when smaller than the symbol size.

As shown in Figure 1A, *B. longum* BB536 can utilize glucose, lactose, and galactose as sole carbon sources, but not fucose and 2′‐FL. When lactose was used as the sole carbon source, after 24 h of incubation, lactose was fully consumed while galactose generated from lactose hydrolysis was excreted in culture media (Table 1). Moreover, the growth of BB536 in galactose was limited (Figure 1A). All bifidobacteria dissimilate carbon compounds anaerobically through the Bifidobacterium shunt pathway (Pokusaeva et al., 2011). Acetic and lactic acids are produced as the end products of sugar fermentation. Besides acetate and lactate, bifidobacteria are capable of producing formate, ethanol, and succinate as fermentation end products to increase the ATP yield (Schöpping et al., 2022). Thus, when glucose, lactose, or galactose were supplied as carbon sources, lactate, acetate, and ethanol were produced as end products by *B. longum* BB536 (Table 1).

**TABLE 1:** Concentration of substrates and extracellular metabolites of *Bifidobacterium longum* subsp. *longum* BB536 and *Bifidobacterium longum* subsp. *infantis* ATCC 15697 in sugar‐free de Man Rogosa and Sharpe media with different carbohydrate sources after 24‐h incubation (g/L).

Strain	Carbohydrate source (10 g/L)	Concentration (g/L) of substrates and metabolites after 24‐h incubation							
		Lactose	Glucose	Galactose	Fucose	2′‐FL	Lactate	Acetate	Ethanol
*B. longum* BB536	Water	0.00	0.00	0.00	0.00	0.00	0.13 ± 0.02	3.80 ± 0.05	0.00
	Lactose	0.00	0.00	2.77 ± 0.28	0.00	0.00	5.68 ± 0.06	5.65 ± 0.28	6.44 ± 0.27
	Glucose	0.00	0.00	0.06 ± 0.00	0.00	0.00	4.53 ± 0.04	8.59 ± 0.05	6.27 ± 0.88
	Galactose	0.00	0.00	8.15 ± 0.74	0.00	0.00	0.54 ± 0.26	4.96 ± 0.33	7.57 ± 1.00
	Fucose	0.00	0.00	0.00	9.78 ± 0.24	0.00	0.19 ± 0.08	3.82 ± 0.32	0.59 ± 0.06
	2′‐FL	0.00	0.00	0.00	0.00	9.33 ± 0.04	0.17 ± 0.05	4.37 ± 0.15	0.38 ± 0.25
*B. infantis* ATCC 15697	Water	0.00	0.00	0.00	0.00	0.00	0.16 ± 0.01	4.05 ± 0.02	2.92 ± 0.19
	Lactose	3.04 ± 0.15	0.00	0.00	0.00	0.00	3.43 ± 0.02	7.68 ± 0.03	3.66 ± 0.15
	Glucose	0.00	0.18 ± 0.10	0.00	0.00	0.00	4.30 ± 0.38	8.49 ± 0.05	4.11 ± 1.31
	Galactose	0.00	0.00	0.25 ± 0.04	0.00	0.00	4.67 ± 0.08	8.92 ± 0.19	5.99 ± 0.63
	Fucose	0.00	0.00	0.00	4.12 ± 0.29	0.00	0.43 ± 0.74	5.00 ± 0.09	0.79 ± 0.16
	2′‐FL	1.42 ± 0.02	0.13 ± 0.01	0.00	0.52 ± 0.10	1.67 ± 0.06	2.75 ± 0.34	7.36 ± 0.02	3.10 ± 0.27

As shown in Figure 1B, *B. infantis* ATCC 15697 showed better growth in glucose, lactose, and galactose compared to BB536, with all OD_600_ values exceeding 1.0 after 24 h of incubation. When lactose was fed as the sole carbon source, galactose was not detected in the culture media (Table 1). Unlike *B. longum* BB536, *B. infantis* ATCC 15697 was able to grow on fucose as a sole carbohydrate source, although the growth is very limited (Figure 1B). This result was reproducible and consistent with previous studies (Crociani et al., 1994; Dedon et al., 2020). Furthermore, we found that ATCC 15697 could utilize 2′‐FL as the sole carbon source (Figure 1B). Lactose, glucose, and fucose were detected in culturing media (Table 1). Similar to BB536, ATCC 15697 produces lactate, acetate, and ethanol as fermentation end products (Table 1).

### 3.2 Changes in pH during fermentation and storage

**FIGURE 2:**
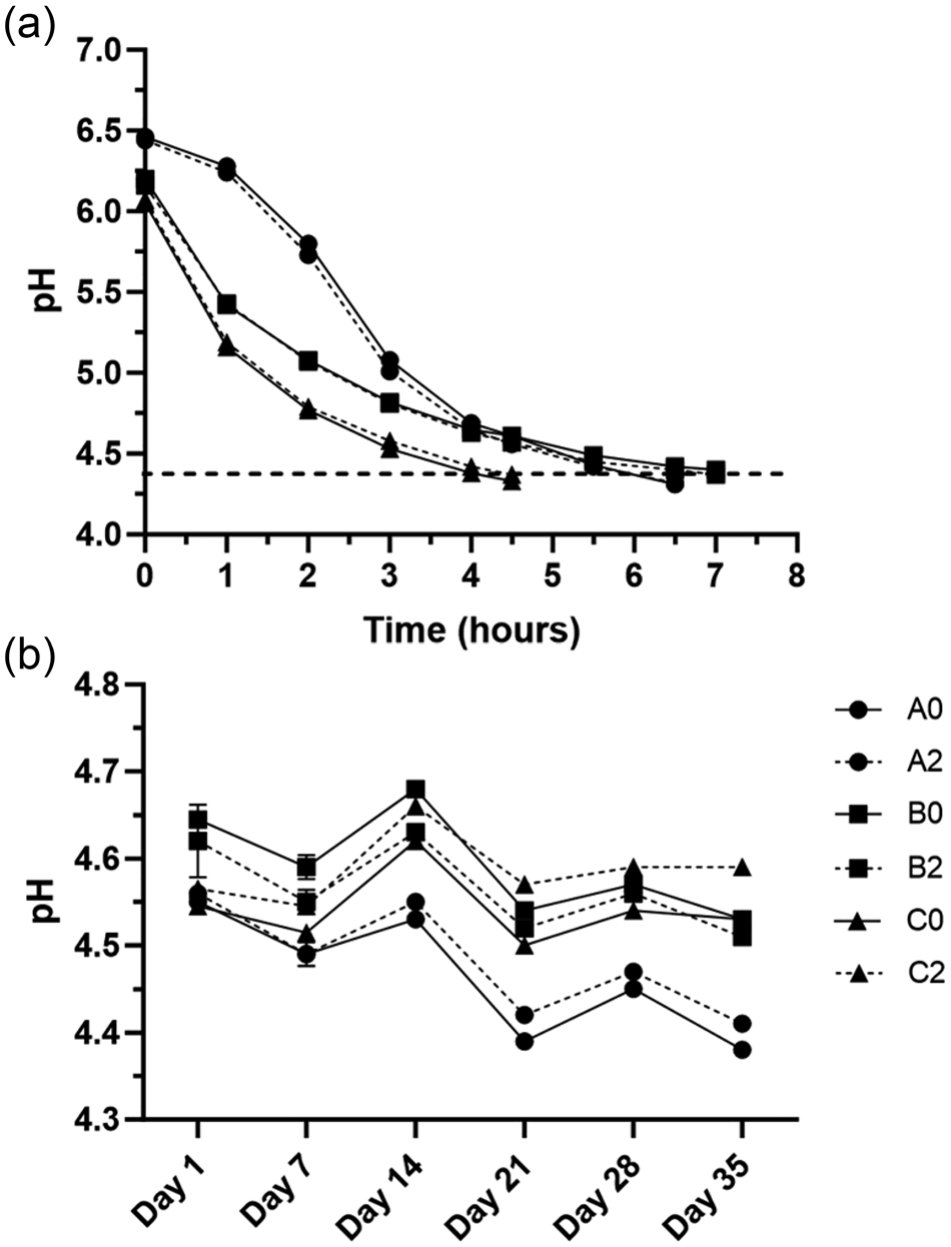
(A) Changes in pH values in six different yogurt samples during fermentation and (B) during refrigerated storage. A0, 0 g/L 2′‐fucosyllactose (2′‐FL) and starter cultures; A2, 2 g/L 2′‐FL and starter cultures; B0, 0 g/L 2′‐FL, starter cultures, and *Bifidobacterium longum* subsp. *longum* BB536; B2, 2 g/L 2′‐FL, starter cultures, and *B. longum* BB536; C0, 0 g/L 2′‐FL, starter cultures, and *Bifidobacterium longum* subsp*. infantis* ATCC 15697; C2, 2 g/L 2′‐FL, starter cultures, and *B*. *infantis* ATCC 15697. Day 1, the day following fermentation; days 7–35, once every week for 5 weeks. Standard errors are not shown due to the lack of data.

The pH changes during fermentation and cold storage were monitored in this study as it is an important processing parameter, particularly from the perspective of yogurt production. The pH values of yogurt samples during fermentation are shown in Figure 2A. The addition of active bifidobacteria strains led to lower initial pH values in Groups B (*B. longum* BB536 added yogurt samples) and Groups C (*B. infantis* ATCC 15697 added yogurt samples) compared to Groups A (only starter culture added yogurt samples). The pH values of Groups B and C dropped significantly at the beginning of the fermentation compared to Groups A. Remarkably, the pH values of Groups C yogurt samples reached the target value of 4.4 within 4.5 h, while it took Groups A and B approximately 6.5 h to reach the same pH value. This can be explained by the better sugar utilization ability of ATCC 15697 (Table 1), which results in higher production of lactic and acetic acids and a faster rate of acidification. There was no significant difference observed within groups (with or without 2′‐FL), indicating that the presence of 2′‐FL would not affect the acidification rate.

The pH values of yogurt samples during cold storage are shown in Figure 2B. Similar to the fermentation results, there was no significant difference observed within groups (with or without 2′‐FL), suggesting that 2 g/L 2′‐FL would not affect the post‐acidification rate and did not have buffering capacity. The average pH value of Groups A was found to be lower than that of Groups B and C. The pH values of Groups A and B slowly decreased during the cold storage. Specifically, the pH value of Groups A dropped from 4.5 to 4.4, and the pH value of Groups B dropped from 4.6 to 4.5. The reduction of pH in yogurt is due to the post‐acidification by acid‐tolerant *L. bulgaricus* by acting upon the remaining lactose content and producing organic acids (Deshwal et al., 2021). In contrast, the pH value of Groups C remained relatively stable around 4.55 during the cold storage.

### 3.3 Viability of mixed starter cultures of *S. thermophilus* and *L. bulgaricus*

In yogurt samples without bifidobacteria (Groups A), cell densities of *S. thermophilus* and *L. bulgaricus* increased to an average of 2.2 log_10_ CFU/g during fermentation (Table 2). During 5 weeks of storage, the mixed yogurt starter cultures, *S. thermophilus* and *L. bulgaricus*, survived at a level well above 10^9^ CFU/g. The high survival of yogurt cultures (10^9^ CFU/g) is consistent with studies showing that *S. thermophilus* and *L. bulgaricus* strains survive well during cold storage at typical yogurt pH. There was no significant difference between Group A0 (without 2′‐FL) and Group A2 (with 2 g/L 2′‐FL) (*p >* 0.05), which indicates that 2′‐FL does not affect the viability of mixed yogurt starter cultures during cold storage.

**TABLE 2:** Viability of *Streptococcus thermophilus* and *Lactobacillus delbureckii* subsp. *bulgaricus* in different yogurt treatments during refrigerated storage (Log_10_ CFU/g).

Day	A0	A2	B0	B2	C0	C2
0	7.10 ± 0.06^a^	7.14 ± 0.15^a^	7.14 ± 0.11^a^	7.19 ± 0.07^a^	7.23 ± 0.09^a^	7.22 ± 0.08^a^
1	9.25 ± 0.02^a^	9.31 ± 0.10^a^	8.70 ± 0.07^b^	8.61 ± 0.17^b^	8.64 ± 0.19^b^	8.57 ± 0.04^b^
7	9.27 ± 0.11^a^	9.22 ± 0.03^a^	8.79 ± 0.11^b^	8.58 ± 0.31^b^	7.60 ± 0.45^c^	7.33 ± 0.16^c^
14	9.40 ± 0.04^a^	9.25 ± 0.19^a^	8.48 ± 0.19^b^	8.44 ± 0.14^b^	7.20 ± 0.10^c^	7.40 ± 0.02^c^
21	9.41 ± 0.05^a^	9.24 ± 0.17^a^	8.47 ± 0.09^b^	8.41 ± 0.11^b^	6.15 ± 0.14^c^	6.07 ± 0.15^c^
28	9.25 ± 0.09^a^	9.18 ± 0.08^a^	8.36 ± 0.09^b^	8.44 ± 0.03^b^	7.34 ± 0.07^c^	7.32 ± 0.04^c^
35	9.15 ± 0.13^a^	9.21 ± 0.16^a^	8.35 ± 0.09^b^	8.33 ± 0.01^b^	6.18 ± 0.23^c^	6.28 ± 0.19^c^

Abbreviations: A0, 0 g/L 2′‐fucosyllactose (2′‐FL) and starter cultures; A2, 2 g/L 2′‐FL and starter cultures; B0, 0 g/L 2′‐FL, starter cultures, and *Bifidobacterium longum* subsp. *longum* BB536; B2, 2 g/L 2′‐FL, starter cultures, and *B. longum* BB536; C0, 0 g/L 2′‐FL, starter cultures, and *Bifidobacterium longum* subsp. *infantis* ATCC 15697; C2, 2 g/L 2′‐FL, starter cultures, and *B*. *infantis* ATCC 15697. Day 0, before fermentation; day 1, the day following fermentation; days 7–35, once every week for 5 weeks.

Values marked with different superscript uppercase letters (a, b, c) indicate that those in the same row are significantly different (*p *< 0.05).

The starter cultures, however, were negatively affected by the addition of BB536 (*p < *0.05), and ATCC 15697 (*p <* 0.05) (Table 2). In yogurt samples containing bifidobacteria (groups B and groups C), *S. thermophilus* and *L. bulgaricus* increased in number by an average of 1.5 log_10_ CFU/g during fermentation (Table 2). The increase of yogurt starter culture cell numbers in Groups B and C was 0.7 log_10_ CFU/g less than the increase of cell numbers (2.2 log_10_ CFU/g) in Groups A, indicating a possible competitive environment in Groups B and C during fermentation. During 5 weeks of storage, the mixed yogurt starter cultures maintained a high CFU (10^8^ CFU/g) in yogurt samples with BB536 (Groups B). However, in ATCC 15697 containing yogurt samples (Groups C), the CFU of the mixed yogurt cultures decreased by 1.4 log_10_ after 1 week of storage, and the final survival level after 5 weeks of storage was around 10^6^−10^7^ CFU/g. Like Groups A, adding 2′‐FL does not affect the viability of the mixed yogurt starter cultures in Groups B (*p *> 0.05) and C (*p* > 0.05) during cold storage.

### 3.4 Viability of bifidobacteria

**FIGURE 3:**
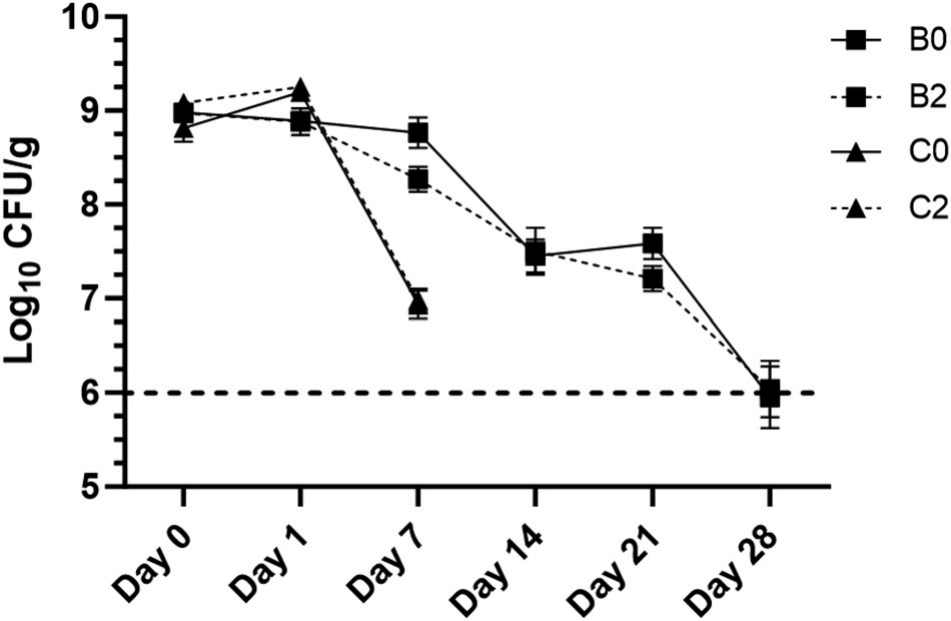
Viability of *Bifidobacterium longum* subsp*. longum* BB536 and *Bifidobacterium longum* subsp. *infantis* ATCC 15697 in different yogurt treatments with and without 2′‐fucosyllactose (2‐FL) during 4°C storage. B0, 0 g/L 2′‐FL, starter cultures, and *B. longum* BB536; B2, 2 g/L 2′‐FL, starter cultures, and *B. longum* BB536; C0, 0 g/L 2′‐FL, starter cultures, and *B. infantis* ATCC 15697; C2, 2 g/L 2′‐FL, starter cultures, and *B. infantis* ATCC 15697. Day 0, before fermentation; day 1, the day following fermentation; days 7–35, once every week for 5 weeks. Results are the average of biological replicates (*n* = 3). Error bars represent standard deviations (SD) and are not displayed when smaller than the symbol size.

BB536 and ATCC 15697 showed decreased survival in comparison to the mixed yogurt starter cultures (Figure 3). In Groups B, BB536 decreased in number by an average of 0.1 log_10_ CFU/g after fermentation. In Groups C, ATCC 15697 increased in number by an average of 0.3 log_10_ CFU/g after fermentation, which can be explained by the better sugar utilization ability of ATCC 15697. During the cold storage, BB536 and ATCC 15697 did not survive in any treatment for the entire duration of cold storage. The average survival above 10^6^ CFU/g was 28 days for Groups B, while for Groups C, it was only 7 days. As per previous investigations, *B. infantis* is more sensitive to storage at 4°C than *B. longum*. Even though ATCC 15697 can utilize 2′‐FL as a carbon source, 2 g/L 2′‐FL did not improve the survival rate of ATCC 15697 (*p >* 0.05). Furthermore, there was no significant difference observed within groups (with or without 2′‐FL), indicating that 2′‐FL does not affect the viability of bifidobacteria.

### 3.5 Changes in lactic and acetic acid contents during cold storage

Organic acids, such as lactic and acetic acids, are generated by the metabolism of microorganisms in yogurt. These acids can increase the shelf‐life of yogurt and also contribute to its unique flavor. In our study, we used HPLC to determine the changes in lactic and acetic acid contents in yogurt samples, as shown in Table 3. The results showed that there was no significant difference (*p *> 0.05) in lactic and acetic acid production between yogurt cultured with or without 2′‐FL.

**TABLE 3:** Concentrations of organic acids (mg/gram per yogurt) in different yogurt samples during refrigerated storage.

Organic acid	Day	A0	A2	B0	B2	C0	C2
Lactic acid	0	0.00^a^	0.00^a^	0.00^a^	0.00^a^	0.00^a^	0.00^a^
	1	9.35 ± 0.40^a^	8.98 ± 0.24^a^	5.24 ± 0.90^b^	5.84 ± 0.61^b^	4.74 ± 0.45^b^	4.65 ± 0.38^b^
	7	9.85 ± 0.20^a^	10.15 ± 0.53^a^	6.19 ± 0.35^b^	6.25 ± 0.36^b^	4.94 ± 0.39^c^	4.86 ± 0.45^c^
	14	10.24 ± 0.18^a^	10.29 ± 0.30^a^	6.68 ± 0.04^b^	6.63 ± 0.38^b^	5.02 ± 0.36^c^	4.92 ± 0.26^c^
	21	10.90 ± 0.21^a^	10.74 ± 0.55^a^	6.13 ± 0.24^b^	6.65 ± 0.28^b^	5.03 ± 0.24^c^	5.27 ± 0.15^c^
	28	10.01 ± 0.13^a^	9.71 ± 0.25^a^	6.15 ± 0.19^b^	6.30 ± 0.70^b^	4.84 ± 0.05^c^	4.73 ± 0.16^c^
	35	10.37 ± 0.46^a^	10.65 ± 0.23^a^	6.61 ± 0.53^b^	6.79 ± 0.26^b^	5.01 ± 0.02^c^	5.01 ± 0.09^c^
Acetic acid	0	0.00^a^	0.00^a^	0.00^a^	0.00^a^	0.00^a^	0.00^a^
	1	0.00^c^	0.00^c^	3.58 ± 0.71^b^	3.76 ± 0.36^b^	5.30 ± 0.48^a^	4.96 ± 0.48^a^
	7	0.00^c^	0.00^c^	4.12 ± 0.07^b^	3.91 ± 0.25^b^	5.40 ± 0.45^a^	5.35 ± 0.48^a^
	14	0.00^c^	0.00^c^	4.29 ± 0.05^b^	4.13 ± 0.23^b^	5.51 ± 0.56^a^	5.38 ± 0.34^a^
	21	0.00^c^	0.00^c^	3.94 ± 0.09^b^	4.00 ± 0.21^b^	5.53 ± 0.20^a^	5.66 ± 0.15^a^
	28	0.00^c^	0.00^c^	3.94 ± 0.07^b^	3.94 ± 0.40^b^	5.25 ± 0.19^a^	5.18 ± 0.26^a^
	35	0.00^c^	0.45 ± 0.01^c^	4.20 ± 0.33^b^	4.08 ± 0.10^b^	5.31 ± 0.01^a^	5.34 ± 0.02^a^

Abbreviations: A0, 0 g/L 2′‐fucosyllactose (2′‐FL) and starter cultures; A2, 2 g/L 2′‐FL and starter cultures; B0, 0 g/L 2′‐FL, starter cultures, and *Bifidobacterium longum* subsp*. longum* BB536; B2, 2 g/L 2′‐FL, starter cultures, and *B. longum* BB536; C0, 0 g/L 2′‐FL, starter cultures, and *Bifidobacterium longum* subsp*. infantis* ATCC 15697; C2, 2 g/L 2′‐FL, starter cultures, and *B*. *infantis* ATCC 15697. Day 0, before fermentation; day 1, the day following fermentation; days 7–35: once every week for 5 weeks.

Values marked with different superscript uppercase letters (a, b, c) indicate that those in the same row are significantly different (*p *< 0.05).

When only yogurt starter culture was added (Groups A), the lactic acid content was around 9 mg per gram of yogurt (mg/g) after fermentation (day 1). Subsequently, lactic acid was produced at a slow pace during refrigerated storage leading to a slow decrease in the pH of Groups A during cold storage. Over the 5 weeks of cold storage, an additional approximately 1 mg/g of lactic acid was cumulatively produced (Table 3). Our yogurt starter culture showed minimal acetic acid production, with only 0.45 mg/g of acetic acid detected on Day 35 in Group A2 (Table 3).

Unlike the yogurt starter cultures, *Bifidobacterium* strains produced lactic and acetic acids as the end products of sugar fermentation in yogurt products, similar to their behavior in pure culture media. As shown in Table 3, BB536 (Groups B) generated around 5.5 mg/g of lactic acid and 3.7 mg/g of acetic acid after fermentation (Day 1). Acetic acid produced by BB536 during fermentation was an important inhibitor of the growth of the *Lactobacillus* strain, resulting in lower viable counts of yogurt starter cultures in Groups B (10^8^ CFU/g) compared to Groups A (10^9^ CFU/g). A slight gradual increase in lactic and acetic acid content was observed throughout the storage period, which could explain the slow decrease in the pH of Groups B during cold storage. Cumulatively, about 1 mg/g of lactic acid and 0.5 mg/g of acetic acid were produced over the 5‐week cold storage.

When ATCC 15697 was added to yogurt samples, around 5 mg/g of lactic acid and 5 mg/g of acetic acid were produced after fermentation (day 1). Compared with Groups A and B, ATCC 15697 produced the highest acetic acid concentration, leading to the lowest viable counts of yogurt starter cultures in Groups C (Table 3). Unlike Groups A and B, the lactic and acetic acid content remained relatively stable during the 5‐week cold storage, which was consistent with the pH results in Section 3.2. The cessation of lactic and acetic acid production could be attributed to the low viable counts of yogurt starter cultures and *B. infantis* ATCC 15697.

### 3.6 Analysis of substrates and metabolites

**FIGURE 4:**
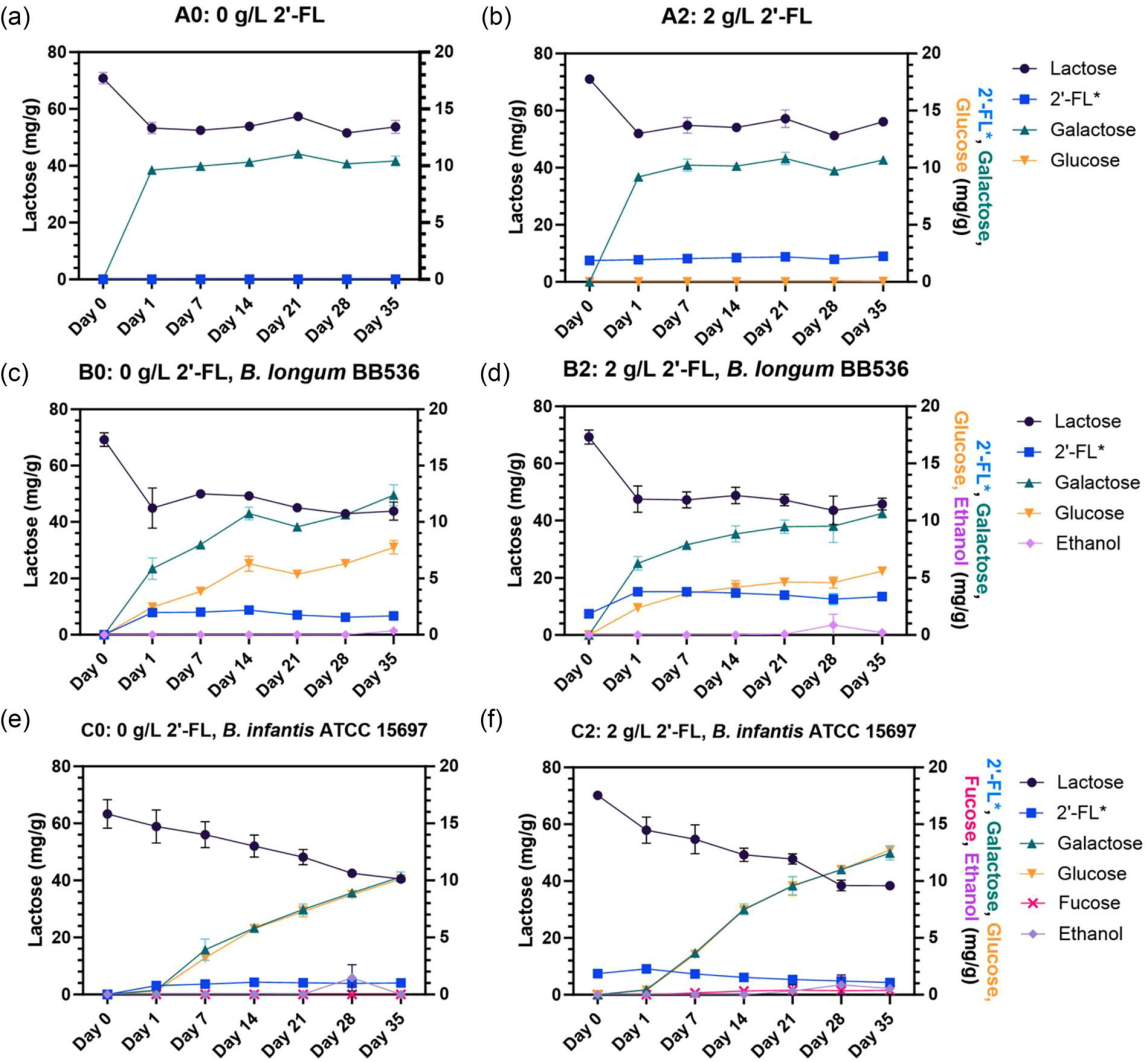
Changes in concentration of substrates and metabolites during 4°C storage. Day 0, before fermentation; day 1, the day following fermentation; days 7–35, once every week for 5 weeks. Results are the average of biological replicates (*n* = 3). Error bars represent standard deviations (SD) and are not displayed when smaller than the symbol size. *, the estimated concentrations of compounds eluting at the retention time of 2′‐FL.

In yogurt samples without bifidobacteria (Groups A), approximately 17 mg/g g of lactose was hydrolyzed, with 10 mg/g of galactose being excreted by the yogurt starter cultures after fermentation (Figure 4A,B). The levels of lactose and galactose remained almost constant during the 5‐week cold storage period (Figure 4A,B). In Group A2 (containing 2′‐FL), 2 mg/g of 2′‐FL remained stable and constant throughout the entire process (Figure 4B).

In yogurt samples containing BB536 (Group B), approximately 20 mg/g of lactose was hydrolyzed, and about 6 mg/g of galactose and 2.5 mg/g of glucose were released after fermentation (Figure 4C,D). During refrigerated storage, lactose was slowly hydrolyzed into galactose and glucose by β‐galactosidase (Figure 4C,D). Interestingly, in both Groups B0 and B2, an estimated 2 mg/g of unknown compounds eluted at the same retention time as 2′‐FL in day 1 samples (Figure 4C,D). Consequently, a total of 2 and 4 mg/g of compounds were identified as 2′‐FL by HPLC in groups B0 and B2, respectively. 2′‐FL and the unknown compounds were stable in yogurt products for at least 5 weeks of cold storage. The unknown compounds possibly could be galacto‐oligosaccharides (GOS). Previous studies have shown that bifidobacterial β‐galactosidase can generate GOS from lactose (Han et al., 2014; Viborg et al., 2014). GOS is primarily composed of disaccharides and trisaccharides with the most representative trisaccharide being galactosyl‐lactose, which is also known to be a minor HMO component (Ambrogi et al., 2021). The production of ethanol was observed starting from day 28, which could be explained by the production of lactic acid, acetic acid, and ethanol by BB536 as fermentation end products.

In yogurt samples containing ATCC 15697 (Groups C), after fermentation, approximately 5 and 12 mg/g of lactose were consumed in Groups C0 and C2, respectively (Figure 4E,F). Unlike Groups A and B, no galactose and glucose accumulation occurred after yogurt fermentation (day 1). This could be explained by the better lactose and galactose utilization ability of ATCC 15697, which led to the complete utilization of galactose and glucose during yogurt fermentation. During cold storage, lactose was continuously hydrolyzed by β‐galactosidase, leading to the excretion of galactose and glucose (Figure 4E,F). Similar to Groups B, in both Groups C0 and C2, estimated 1 mg/g of unknown compounds eluted at the same retention time as 2′‐FL in day‐1 samples (Figure 4E,F). Consequently, a total of 1 mg/g and 3 mg/g of compounds were identified as 2′‐FL by HPLC in Groups C0 and C2, respectively. Remarkably, in Group C2, a decline in the level of these compounds was observed starting from day 7, accompanied by the production of approximately 0.4 mg/g fucose, which was consistent with pure culture media results in Section 3.1. This phenomenon is attributed to the ability of ATCC 15697 to utilize 2′‐FL as a carbon source. Similar to Groups B, the production of ethanol was observed starting from day 28, which could be explained by the production of lactic acid, acetic acid, and ethanol by *B. infantis* ATCC 15697 as fermentation end products.

## 4 CONCLUSION

This study has shown the impact of 2′‐FL on the viability of both traditional yogurt starter cultures and probiotic bifidobacterial strains in yogurt. Our results indicate that 2′‐FL remains stable and constant in yogurt products for at least 5 weeks, and the addition of 2′‐FL does not significantly alter yogurt properties, including fermentation parameters, associated metabolites, and the viability of mixed yogurt starter cultures and bifidobacterial strains. This suggests that it is promising to add 2′‐FL into yogurt products as a prebiotic. Unlike yogurt containing only starter cultures, the properties of yogurt with bifidobacteria change throughout refrigerated storage. The addition of bifidobacteria had a negative impact (*p* < 0.05) on the survival rate of yogurt starter cultures, *S. thermophilus* and *L. bulgaricus*, attributed to the production of acetic acid by bifidobacteria. Moreover, it is difficult to maintain a high survival rate of bifidobacterial strains in final yogurt products because of their sensitive nature, and 2 mg/g 2′‐FL could not enhance the survival of bifidobacterial strains in yogurt during cold storage. *B. longum* BB536 survived at a level higher than 10^6^ CFU/g for 28 days, while only 7 days for *B. infantis* ATCC 15697. In yogurt samples with bifidobacteria, lactose was slowly hydrolyzed by β‐galactosidase during cold storage, leading to the excretion of galactose and glucose. This could potentially enhance the sweetness of yogurt products.

## AUTHOR CONTRIBUTIONS

**Zifan Xie**: Methodology; writing—original draft; writing—review and editing; conceptualization; data curation; investigation. **Chanwoo Kim**: Methodology; writing—review and editing. **Michael J. Miller**: Writing—review and editing; supervision; conceptualization; resources. **Yong‐Su Jin**: Writing—review and editing; funding acquisition; supervision; conceptualization; resources.

## CONFLICT OF INTEREST STATEMENT

The authors have no conflicts of interest to declare. All co‐authors have seen and agreed with the contents of the manuscript, and there is no financial interest to report. We certify that the submission is an original work and is not under review at any other publication.
